# Women in Surgery Events Alone do not Change Medical Student Perceptions of Gender Bias and Discrimination in Orthopaedic Surgery

**DOI:** 10.3389/fsurg.2022.905558

**Published:** 2022-05-25

**Authors:** Bethany Hull, Olivia Pestrin, Caitlin M. Brennan, Rosie Hackney, Chloe E.H. Scott

**Affiliations:** ^1^ Edinburgh Medical School, University of Edinburgh, Edinburgh, United Kingdom; ^2^ Edinburgh Orthopaedics, Department of Trauma and Orthopaedic Surgery, Royal Infirmary of Edinburgh, Edinburgh, United Kingdom

**Keywords:** gender, women in surgery, diversity & inclusion, medical students, discrimination, bias, orthopaedic surgery

## Abstract

**Aims:**

This study investigated the perceptions of medical students regarding the barriers to pursuing a career in trauma and orthopaedics (T&O); and whether these perceptions were altered by attending an event promoting women in T&O.

**Methods:**

An event consisting of presentations and interactive sessions from two female T&O trainees was hosted online. Attendees completed pre and post-event questionnaires. Students were asked about their previous exposure to T&O, perceptions of gender imbalances in T&O and what barriers they perceived prevented women from entering T&O. Univariate analysis was performed to identify changes in perceptions following the event.

**Results:**

Pre-event questionnaires were completed by 102 people; and post-event by 52. Although 64/102 respondents were considering a career in T&O, 26/102 were dissuaded by perceived gender disparities. Perceptions of gender disparities were significantly higher in UK based attendees compared to other nationalities (*p* = 0.047). Attendees were more likely to want to pursue a career in T&O if they had been directly exposed at medical school (*p* = 0.044), but exposure did not alter perceptions of women in T&O. The most common perceived barrier was the orthopaedic stereotype followed by male dominated workplace culture, and lack of female role models. Pre and post-event responses did not differ significantly for any areas examined.

**Conclusion:**

There are significant concerns amongst medical students regarding gender based discrimination within T&O, and these perceptions were not altered by attending a one-off women in T&O event. Early exposure to T&O appears important to improve interest in orthopaedics, whereas negative stereotyping is a barrier.

## Introduction

Since 1996 medical school cohorts in the United Kingdom have consistently been made up of more females than males, yet this has not translated to the surgical workforce (1). Over the past 15 years, the percentage of women in surgical training has increased by only 3.9% (2). Currently 14.5% of consultant surgeons within the National Health Service [NHS] are female. Female representation within trauma and orthopaedics (T&O) is the lowest of any surgical speciality; 6.5% of NHS T&O consultants and 18.7% of speciality registrars identify as female (3). Although the number of women in training is increasing, efforts to both recruit and retain female trainees is required.

A recent systematic review of gender bias and sexual discrimination in orthopaedics found strong evidence that gender based sexual discrimination is unfortunately widespread within the speciality, significantly impacting women at all stages of their careers (4). There has been limited research into whether women are deterred from applying for T&O, and the causes of this. Issues that have been cited include minimal exposure to musculoskeletal topics during medical school, lack of female role models, discriminatory recruitment, male dominance within the speciality, the perception of requiring physical strength, and concerns over the length of training and ability to have a family (5–9).

Experiences during medical school are thought to be the most common reason for choosing a career in a given speciality and therefore medical students are an important population to investigate and potentially target (10). The aims of this study were to investigate the perceptions of medical students’ regarding pursuing a career in T&O and whether these perceptions and beliefs were altered by attending an online event promoting women in T&O.

## Methods

A collaborative online event entitled “Women in Trauma and Orthopaedics” was created and organised by the Association of Women Surgeons (AWS) Edinburgh Chapter and Edinburgh University Trauma and Orthopaedic Society (EUTOS). The event was designed to facilitate discussion about women in T&O and encourage female medical students interested in the profession. It was advertised on Facebook through EUTOS and AWS and was open to anyone. The event was of 1 h duration and included speeches from two Edinburgh based T&O trainees, followed by a question and answer session. The speakers shared their career paths to date, experiences of being a woman in T&O and gave advice for students interested in T&O.

Before and after the event students were asked to complete a survey that was distributed via Facebook and email. Students provided demographic data including year of medical school training, location of medical school and gender identity. Students were asked about their exposure to T&O to date, perceptions of gender imbalances in T&O, whether they were dissuaded from applying because of gender imbalances, and what barriers they identified as preventing women from entering T&O. The majority of questions were asked on a five point Likert scale. The pre and post-event surveys may be found in appendix A.

Statistical analysis was performed using SPSS Version 25.0. Categorical variables were compared using Chi squares or Fisher’s exact test. Continuous variables were examined for normality: parametric data was compared between groups using an unpaired students T-test. A *p* value of <0.05 was considered statistically significant.

## Results

Pre-event questionnaires were completed by 102 students; 52 of whom completed the post-event questionnaire. Consent was obtained from individuals to analyse their anonymised data.

### Pre-Event Questionnaire

Pre-event questionnaires identified that 98/102 (96%) of respondents identified as female and 89/102 (87%) were current medical students. The remainder had already graduated but were in the early years of postgraduate medical training before specialty training. Almost two thirds (64/102, 63%) were strongly considering a career in orthopaedics. One third of attendees (33/102, 32%) reported little or no exposure to T&O as part of their medical education or training to date. Thirty percent (30/99) had undertaken research in T&O. One fifth (20/96) considered that they were at a point where they could apply to T&O training.

**Figure 1:**
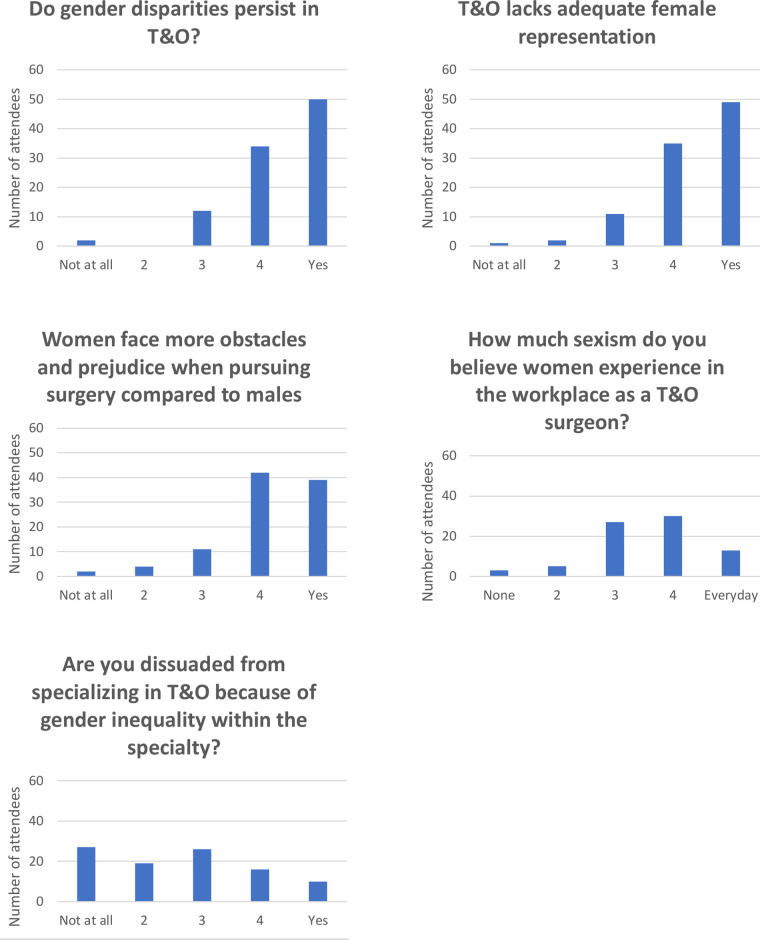
Pre-event perceptions of T&O.

Perceptions of T&O as recorded on the pre-event questionnaire are given in Figure 1. A quarter of attendees (26/102) reported pre-event that they had been dissuaded from entering T&O by the perceived gender disparities within it.

Responders were international though the majority 68/102 (67%) were based in the United Kingdom. Geographic base did not significantly affect the responses to any of the questions regarding women in T&O: the number strongly considering a career in orthopaedics (*p* = 0.415, Chi square); the proportions who felt that orthopaedics lacks adequate female representation (*p* = 0.327, Chi square); that women face more obstacles and prejudice when pursuing surgery compared to males (*p* = 0.130, Chi square); the amount of sexism experienced by women in the workplace (*p* = 0.106, Chi square); the amount of exposure to T&O as a specialty (*p* = 0.600, Chi square) or the number dissuaded from entering T&O due to gender inequality within the specialty (*p* = 0.376, Chi square). There was a significant difference in the perception of gender disparities within orthopaedics between UK and non-UK attendees (*p* = 0.047, Chi square): UK 59/66 (89%) vs non-UK 25/30 (83%) were strongly aware of gender disparities.

**Figure 2:**
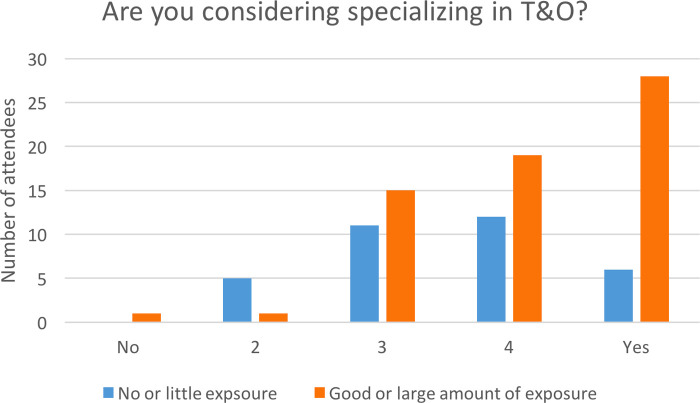
Association between desire to specialise in T&O and exposure to T&O.

Direct exposure to T&O was significantly associated with a desire to pursue a career in T&O: 47/64 (73%) with direct exposure were considering a career in orthopaedics compared to 18/34 (52%) with little or no exposure (*p* = 0.044, Chi square) (Figure 2). Exposure to T&O did not however affect perceptions of gender disparities (*p* = 0.766, Chi square); lack of female representation (*p* = 0.340, Chi square); lack of role models (*p* = 0.135, Fisher’s exact); the perception of more obstacles for women (*p* = 0.181, Fisher’s exact); or the perception of how much sexism women face at work (*p *= 0.645, Chi square). Importantly, previous direct exposure to T&O did not significantly affect the number of students dissuaded from a career in T&O due to gender disparities within it: 13/45 (29%) with direct exposure had been dissuaded vs 13/27 (48%) with no direct exposure (*p* = 0.100, Chi square).

**Figure 3:**
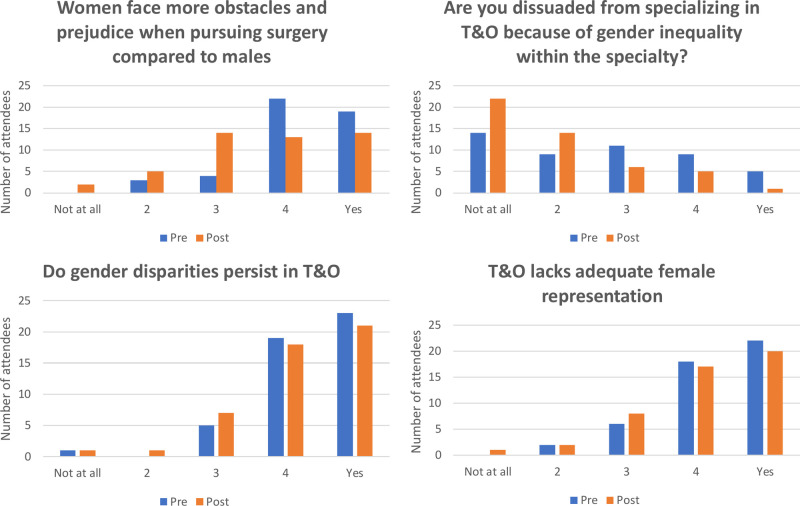
Pre and post event perceptions of T&O compared.

### Post-Event Questionnaire

Pre and post event responses to the statements regarding women in T&O did not differ significantly for any of the areas examined (Figure 3): Orthopaedics lacks adequate female representation (*p* = 0.751, Chi square); Women face more obstacles and prejudice when pursuing surgery compared to males (*p* = 0.078, Fisher’s exact); Are you dissuaded from specialising in trauma & orthopaedics because of gender inequality within the specialty? (*p* = 0.097, Fisher’s exact); Do gender disparities persist in orthopaedic surgery? (0.758, Chi square).

The most common perceived barrier to women entering a career in T&O on post-event questioning was the orthopaedic stereotype followed by concerns regarding male dominated workplace culture and a lack of female role models (Table 1). Attendees identified a mean of 4.3 (SD 2.0) barriers to entering a career in T&O from a maximum possible of 8 (Table 1). The mean number of perceived barriers was significantly higher in the UK (*n* = 34) compared to non-UK (*n* = 14) attendees: mean difference of 1.60 (0.46 to 2.74 95%CI, *p* = 0.007 Unpaired T-test). Previous exposure to T&O (*n* = 30) did not affect the mean number of perceived barriers compared to no or little previous exposure (*n* = 19): mean difference 0.45 (−0.75 to 1.64, *p* = 0.456, unpaired T-test).

**Table 1:** Perceived barriers to women entering a career in orthopaedics identified in the post-event questionnaire (*n* = 52).

Perceived Barrier	*N* [%]
The orthopaedic stereotype	36 [69]
Male dominated workplace culture	33 [63]
Lack of role models	32 [62]
Concerns about having a family causing a career lag compared to men	29 [56]
Sexist comments at work	28 [54]
Work/life balance	24 [46]
Patriarchy	17 [33]
Poor exposure at medical school	15 [29]

## Discussion

The medical students and early years trainees who attended the web based women in orthopaedic surgery event reported high levels of concerns regarding gender bias and discrimination within T&O both before and after attending the webinar. Perceptions of gender disparities were significantly higher in UK based attendees compared to attendees of other nationalities. Though attendees were significantly more likely to want to pursue a career in T&O if they had been directly exposed to T&O at medical school, this exposure did not alter their perceptions of women in T&O for any of the variables examined. Though all attendees were interested in careers in surgery, and two-thirds were strongly considering a career in T&O, in the pre-event survey 28% of attendees stated that they had been dissuaded from entering T&O by the perceived gender disparities within it.

These concerns are not unfounded. A UK study in 2009 showed 13% of 54 orthopaedic surgeons felt that women are incapable of operating, with 5.6% believing women should purposefully be pressured into leaving surgery, and 21% saying a woman’s family responsibilities should not be accommodated in surgery (11). A recent survey of 81 female surgeons in the UK found that 53% perceived orthopaedics to be a sexist speciality, which was 40% more than any other surgical speciality (12). It is unknown if these views were from surgeons with first hand experience of working in T&O. A questionnaire of 96 female medical students and junior doctors in two UK hospitals in 2012 found 15% of medical students and 18% of foundation doctors felt they had been subject to sexual discrimination in the surgical workplace. One example of a comment to a female medical student by an orthopaedic consultant was “6 girls?! That’s at least 3 full time doctors between you …” (13).

Compared to other international students and early years trainees, the perception of gender disparities was higher among UK students, who also perceived significantly more barriers to women pursuing careers in T&O. To the authors knowledge there is no published research into differences in perceptions of gender disparity in orthopaedics between countries, however Marks et al. carried out a global survey of 639 medical students from 75 countries on more general perceptions of surgery. They found women from lower income countries and lower middle income countries were 40% less likely to consider a career in surgery than men, when controlling for other factors (14). Our data found geographic location did not significantly affect the number considering a career in orthopaedics, however we did not divide countries according to their income.

This study found direct exposure to T&O was significantly associated with a desire to pursue a career in T&O. This highlights the importance of ensuring female medical students and junior doctors have adequate exposure to the speciality to stimulate interest, a concept which is supported by existing literature. A 2001 survey of 122 US medical schools found compulsory musculoskeletal medicine teaching was associated with a 12% higher rate of application to orthopaedic residency programmes among all students (15). The relative difference was greater among women with a 75% increase in application rates (15).

A review conducted by O’Conner indicated that interest in orthopaedics often begins before medical school and actions to address gender imbalance in T&O needs to be implemented at the earliest stages of training (16). An example of this is the Perry initiative started in the US in 2012, designed to inspire young women to become leaders in orthopaedics by running hands-on outreach programs for female high school, college, and medical school students. A study of 88 first and second year medical students enrolled in the Perry outreach programme had very positive results. The curriculum consisted of 90 min of hands-on mock orthopaedic surgery, and 90 min of lectures from female orthopaedic surgeons addressing the following topics: stereotypes and misconceptions about orthopaedic practice, orthopaedic subspecialties, academic and prior experience expectations for residency application, and work-life balance. The programme positively influenced women to choose T&O as a career and programme participants had a high match rate to orthopaedic residency. Importantly the programme also significantly improved perceptions of lifestyle, workforce diversity, and the physical demands of orthopaedics (17).

The influence of exposure to T&O was investigated further in a prospective study by Baldwin et al. (9). They aimed to report female medical students’ attitudes towards orthopaedic surgery compared to male students, the factors that make orthopaedics more and less appealing to students, and whether educational resources can be used to increase students’ interest. A programme of orthopaedic lectures and electronic resources were distributed regularly to 154 medical students throughout the study period. Personal, independent and school exposures were all significantly related to baseline interest among women. Before the programme began men were more likely to be interested in orthopaedics than women, however, following the programme this predisposition was removed. The programme also improved women’s perceptions on their ability to succeed in orthopaedic residency interviews. Unlike the Perry initiative, the programme did not change how deterred women felt by the perceived physical demand of operations nor their perceptions of their gender inhibiting career progression or opinions on male domination within the speciality. This is in keeping with our findings. Despite T&O exposure increasing students desire to pursue a career in T&O, exposure did not significantly affect the perceptions of gender bias and discrimination within the speciality in the current study. It also did not reduce the number of students dissuaded from a career in T&O due to gender disparities within it. This suggests exposure to T&O should be focused on reducing misconceptions and stereotypes as well as offering hands-on experience.

The need to dispel stereotyping is further supported by this study, which identified orthopaedic stereotyping as the most common perceived barrier to women entering orthopaedics. Studies show that negative stereotyping of specialities within medical schools has an impact on career choice and often occurs in early years (18). Maidment et al. suggest medical schools should proactively provide information and career advice as part of undergraduate education to combat negative propaganda and encourage students to choose careers that suit them (19). Gender stereotypes are particularly dangerous and discourage a balanced workforce. One study showed that lack of ambition or concern over long working hours and family life were not dissuading factors to women pursuing a surgical career, but rather perceptions about their ability to succeed were. If surgery is consistently stereotyped as a male speciality and this is reinforced by exposure to predominantly male consultants, it is harder for women to see themselves succeeding in the field (20). Therefore, those currently working in the field, in particular leaders, need to make effort to encourage women and challenge any negative stereotyping that they encounter in the workplace. This will help to create an inclusive culture and prevent perpetuation of negative stereotypes.

After stereotypes, the second and third most highly ranked barriers by respondents were a male dominated workplace culture and a lack of role models. It is consistently documented that females value role models of the same sex more than males. In a survey of 205 medical students in the US 33% of female respondents ranked role models of the same sex as an important factor in speciality choice, as opposed to 9% of males (5). A 2013 survey of 529 orthopaedic residents also found significantly more women than men believed having a role model of the same sex or ethnicity was a positive factor to enter orthopaedics (59% vs 25%) (21).

The current study found that a singular event with presentations from two positive female role models did not significantly affect participants’ perceptions of women in T&O and the challenges they experience. This implies that female role modelling cannot be delivered simply by one off remote events. Perhaps a more personal experience or longer term exposure to visible female T&O surgeons is required to change such opinions. Some studies have cast doubt on the benefit of female role models. Kerr et al 2016 survey of 96 female medical students and foundation doctors found while 58% had encountered a female surgical role model during their career, only seven of these felt this had strongly influenced their career decision, and approximately 30% stated they had been dissuaded from a career in surgery by the interactions (13). This is potentially due to female surgeons sharing their challenging career experiences and discouraging respondents. Alternatively one student thought female surgeons are “anti-female”, and can be unsupportive or inattentive. This highlights the need to ensure not only visibility of same sex role models, but also the availability of positive, supportive, and affirmative counselling for women by surgeons male or female. Furthermore, a study of 101 US medical schools found no correlation between the average number of female orthopaedic faculty at an institution and the total number of female orthopaedic applicants from that institution (22). A US survey of 238 first year medical students’ perceptions of surgeons found that females had a less favourable view of surgeons than their male counterparts and concluded recruitment may be improved by surgeon educators using a “communal demeanour” in their interactions with students, regardless of the students gender or interest in surgery (23). This again supports the importance of having encouraging role models either male or female. These studies indicate the complexity of the issue and the need to further investigate ways to encourage female medical students and establish consistent positive female leadership.

Limitations of this study include that it was not limited to medical students, however non-medical student attendees were all in their early post-graduate years. The results may not be generalisable to the medical student population, but reflect those of medics at a stage before they have committed to a specialty. A further limitation is that the survey was written by the authors and was not validated prior to its use.

Only 52/102 of the participants who filled out the pre-event survey filled out the post-event survey, however this was due to 50 people who completed the pre-event survey not attending the event. This study is vulnerable to self-selection bias as those attending the event and filling out the survey may have had more strongly held views regarding women in T&O than the average medical student. This is a common issue in studies of this nature as investigated initiatives are often voluntary and require pre-existing interest. Study of a more representative group of medical students would be a useful future step. A recent survey of 27 US medical schools attempted this by surveying students before and after their orthopaedic rotation, however the response rate was low at 26%. Compared with before their rotation, after their rotations women believed less that they would have to work harder than others to be valued equally on the rotation and thought orthopaedic surgery friendlier, more diverse and less sexist. However, they were still less likely than men to want to pursue a career in orthopaedic surgery (24). This suggests that, contrary to our study and the study by Baldwin et al., but in keeping with the Perry initiative, orthopaedic exposure may be valuable in improving perceptions of orthopaedics. However, it appears there is still something further than increased exposure which needs to be done to encourage women into the profession.

To the authors knowledge there are currently no UK based studies focusing on medical student perspectives on orthopaedics or how these perspectives might be altered. One small UK study investigating factors increasing the flow of students to vascular surgery found a one day student surgical society conference attended by 36 students, resulted in a 18% decrease in the negative perception that vascular surgery is female-unfriendly and 33% increase in interest in vascular surgery (25). However, the gender of respondents was not available making these results hard to interpret in the context of improving female representation.

T&O must work to achieve a greater level of female representation by further investigating the barriers faced by women, and potential solutions. It is crucial to diversify the T&O workforce not only to allow equal opportunities for men and women but to have a workforce that can generate diverse perspectives, greater degrees of innovation and better understanding of the patient population; and therefore provide the highest level of patient care (26).

In conclusion, this study established that medical students interested in surgery and T&O believe there are a number of barriers preventing women from pursuing a career in T&O surgery, and that these beliefs were not altered by attending a single women in orthopaedics event. Early exposure to T&O appears to be an important factor in increasing interest in orthopaedics whereas negative stereotyping is a barrier. This indicates that work should be targeted early in medical school to prevent such stereotypes from being ingrained in medical students. The culture that has lead to these negative stereotypes must continue to be challenged. A lack of same sex role models was also identified as a barrier and efforts need to be made to provide positive and encouraging support to women in medical school in order to eliminate established negative perceptions and cultivate a more welcoming and supportive work environment. Initiatives that are able to actively challenge negative perceptions and stereotypes whilst also providing exciting hand-on exposure to the specialty may prove to be the most successful in improving female interest in orthopaedics.

## Data Availability

The raw data supporting the conclusions of this article will be made available by the authors, without undue reservation.
